# The Unique Neonatal NK Cells: A Critical Component Required for Neonatal Autoimmune Disease Induction by Maternal Autoantibody

**DOI:** 10.3389/fimmu.2014.00242

**Published:** 2014-05-28

**Authors:** Claudia Rival, Yulius Setiady, Eileen T. Samy, Jessica Harakal, Kenneth S. K. Tung

**Affiliations:** ^1^Departments of Pathology and Microbiology, Beirne Carter Center for Immunology Research, University of Virginia, Charlottesville, VA, USA; ^2^ImmunoGen Inc., Waltham, MA, USA; ^3^EMD Serono Research Institute, Inc., Billerica, MA, USA

**Keywords:** NK cells, Ly49 receptors, neonatal immunology, immune complex, autoimmune ovarian disease, regulatory T cells, congenital heart block, neonatal viral immunity

## Abstract

Human maternal autoantibodies can trigger autoimmune diseases such as congenital heart block (CHB) in the progeny of women with lupus or Sjogren’s disease. The pathogenic effect of early autoantibody (autoAb) exposure has been investigated in a murine neonatal autoimmune ovarian disease (nAOD) model triggered by a unique ZP3 antibody. Although immune complexes (IC) are formed in adult and neonatal ovaries, ZP3 antibody triggers severe nAOD only in <7-day-old neonatal mice. Propensity to nAOD is due to the uniquely hyper-responsive neonatal natural killer (NK) cells that lack the inhibitory Ly49C/I receptors. In nAOD, the neonatal NK cells directly mediate ovarian inflammation and oocyte depletion while simultaneously promoting *de novo* pathogenic ovarian-specific T cell responses. Resistance to nAOD in older mice results from the emergence of the Ly49C/I^+^ NK cells that regulate effector NK cells and from CD25^+^ regulatory T cell control. In preliminary studies, FcγRIII^+^ NK cells as well as the ovarian resident FcγRIII^+^ macrophages and/or dendritic cells were found to be as indispensable players. Activated by ovarian IC, they migrate to lymphoid organs where NK cell priming occurs. Remarkably, the findings in nAOD are very similar to those reported for neonatal responses to a retrovirus and its cognate antibody that lead to long-lasting immunity. Studies on nAOD therefore provide insights into maternal autoAb-mediated neonatal autoimmunity, including CHB, while simultaneously uncovering new properties of the neonatal innate and adaptive responses, lethality of premature infant infection, and novel neonatal antiviral vaccine design.

## Human Neonatal Autoimmune Disease Induction by Maternal Autoantibody Exemplifies Neonatal Propensity to Autoimmunity

Increased susceptibility of premature human infants and neonatal mice to infections is well-known (1). What is less appreciated is that neonatal mice are also more susceptible to autoimmune disease. For example, spontaneous autoimmune diseases occur in mice thymectomized between days 1 and 4 but not after day 7 of life (2). Autoimmune diseases induced by tissue antigen or peptide immunization require complete Freund’s adjuvant (CFA) in adult but not in neonatal mice (3–6). Indeed, neonatal female mice immunized with an auto-peptide from the gender-specific ovarian zona pellucida 3 (pZP3) antigen, mounted peptide-specific T cell responses, and developed autoimmune ovarian disease (AOD) in 3 weeks. In contrast, the same ZP3 peptide induced tolerance in neonatal male mice as a foreign antigen (4). The opposing responses to self and foreign antigens in the neonates suggest that the neonatal tolerance paradigm (7, 8) should be revisited because it is built mainly on responses to foreign antigens or peptides including alloantigen (9–15). Indeed, in contrast to the neonatal tolerance paradigm, newborn mice responded as efficiently as adult mice to viral, nominal, and autoantigen, when the antigen dose, injection site, and adjuvant type were adjusted (16–18). Finally, neonatal propensity to autoimmunity is supported by the induction of fetal or neonatal self-tissue damage after the transplacental transfer of maternal autoantibody (autoAb) that often does not harm the adult.

Autoimmune disease occurs preferentially in women of reproductive age, and circulating autoAb is a hallmark of autoimmunity. The finding of neonatal autoimmune disease caused by maternal autoAb establishes its pathogenic potential. The impact of maternal autoAb is often transient, and neonates recover as antibody level declines. However, in some cases, maternal autoAb effects persist and induce permanent tissue damage. An example is congenital heart block (CHB) that occurs in the fetuses or infants of women with systemic lupus erythrematosus (SLE) or Sjogren’s disease. Although CHB has many possible etiologies (19), a strong candidate is the transplacental transfer of maternal autoAb against the ribonucleoproteins SSA/Ro and SSB/La (20), with SSB/La having a stronger association (21). The maternal autoAb damages the atrioventricular node of the cardiac conduction system by an unknown mechanism. Likely, CHB pathogenesis involves excessive apoptosis of cardiomyocytes, tissue inflammation and fibrosis, and the interaction of macrophages and fibroblasts (21). The clinical impact of CHB is significant. Although about 2% of neonates from mothers with autoAb to SSA/Ro and SSB/La develop CHB (20), the recurrence rate for an autoAb-positive mother with a previously affected child is 16–18% (22). Importantly, complete or third degree CHB is irreversible with a mortality rate of 12–43% (22). Although first or second degree heart block may reverse with treatment, most children require permanent pacemakers (22).

Maternal autoAb can induce neonatal autoimmune diseases other than CHB. Neonatal myasthenia gravis, associated with severe deformity and difficult deliveries, is caused by maternal autoAb to the fetal form of acetylcholine receptor and a muscle-specific kinase (23, 24). Neonatal Graves’ disease involves agonist autoAb targeting the thyroid stimulating hormone receptor (19). Neonatal pemphigus is mediated by autoAb to the desmoglein-1 or 3 antigens (25, 26). Several studies have shown a positive correlation between anti-phospholipid or lupus anticoagulant autoAb, present in patients with SLE or anti-phospholipid syndrome, and a high risk of premature onset of labor, low birth weight and early miscarriages of their progeny (19, 27). In addition, neuronal apoptosis and subsequent abortion are consequences of maternal autoAb to DNA in SLE patients (28); and autism spectrum disorders are linked to maternal autoAb against fetal brain antigens (29, 30). The mechanism of human neonatal autoimmunity is currently uncertain, and we have used the neonatal autoimmune ovarian disease (nAOD) model to address the questions of why neonates are more susceptible to autoimmune disease, and how maternal autoAb induce the tissue damage. Below, we have described some of the characteristics and advantages of nAOD that make it a very useful model.

In addition to its ability to cause tissue injury and disease, antibody has been found to elicit changes in neonatal or very young mice and promote subsequent full-blown autoimmune diseases. An example of the “pre-disease” state is the pre-diabetes that precedes clinical diabetes by months to years (31–34). In NOD mice, a very early cascade of cellular and cytokine responses to local immune complexes (IC) in pancreatic islets was found to cause subsequent development of pathogenic T cell responses and clinical juvenile diabetes (35). In addition, maternal autoAb directed to islet antigens was reported as a requirement for type I diabetes in the NOD mice (36) or to accelerate diabetes onset in a transgenic mouse model (37).

Therefore, maternal autoAb can induce either transient pathology or more persistent tissue damage in the progeny. It may also condition the fetus/infant early in life for a late onset autoimmune disease. In these situations, the design of novel preventive therapies will necessitate a thorough understanding of the pathogenic autoimmune response in the very young individuals, including their innate and adaptive neonatal responses to IC created by autoAb.

## Neonatal Autoimmune Ovarian Disease Model and Its Unique Features

The aforementioned findings indicate that newborns are simultaneously more susceptible to infections and to autoimmunity. While “immaturity” of the neonatal immune system might explain the increased sensitivity to pathogens, it is not immediately apparent why newborns are more susceptible to autoimmune disease. A potential explanation is that the maturing neonatal immune system is less stringently regulated relative to the adults. Thus, an overactive neonatal response would induce both autoimmune disease and severe post-infection immunopathology, including sepsis. While other authors support the latter (38), our study focuses on the questions of why newborns are more susceptible to nAOD and how nAOD is induced. We have summarized our results in this review.

Autoimmune ovarian disease is a known cause of primary or secondary premature ovarian failure that can lead to infertility of pubertal and adult women (39, 40). Because ovarian dysfunction is not generally manifested until puberty, it cannot be certain that primary AOD, like type I diabetes, is preceded by a “pre-disease” exemplified by nAOD. While the clinical relevance of nAOD remains unresolved, the murine nAOD model itself has proven to be an excellent platform for investigating the role of the neonatal immune response to autoantigen and the role of autoAb in autoimmune disease pathogenesis.

**Figure 1:**
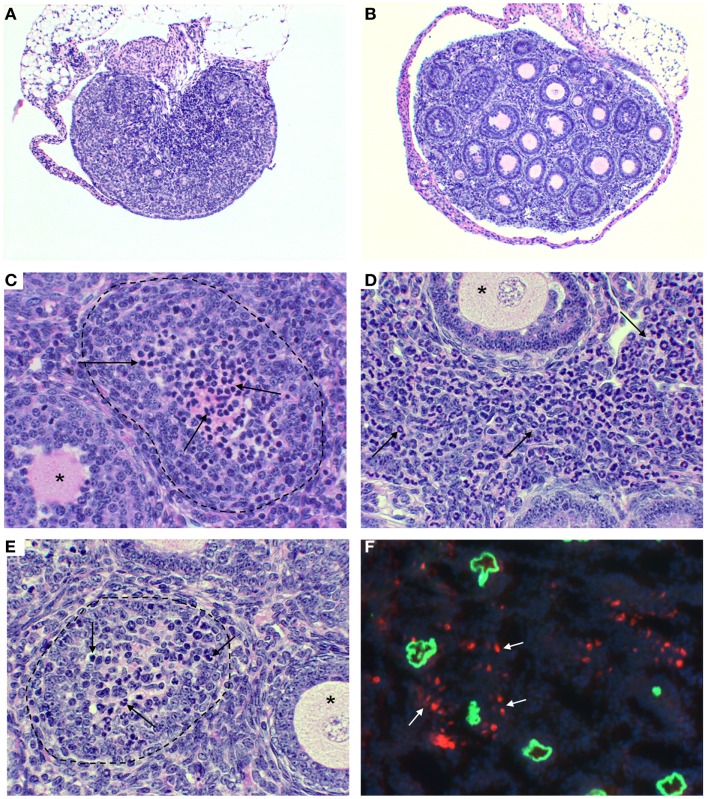
**Ovarian immunopathology of TI-nAOD in C57BL/6 Rag1^−/−^ mice injected with pZP3 monoclonal antibody (mAb) in the first week of life**. **(A)** Ovarian atrophy with major oocyte depletion and disrupted ovarian architecture. **(B)** Normal ovarian histology in mice injected with ZP3 mAb and NK cell-depleting (NK1.1) mAb. **(C–E)** Mononuclear cell-dominant **(C**, arrows), or granulocyte-dominant **(E**, arrows) infiltrates inside ovarian follicles is a common feature of ovarian pathology in TI-nAOD. The dotted lines **(C,E)** outline ovarian follicles where oocytes are replaced by inflammatory cells while the asterisks **(C–E)** indicate normal ovarian follicles. **(D)**, Inflammatory cells also infiltrate the ovarian interstitium (arrows). In **(F)**, Immunofluorescence detection of mouse IgG (green fluorescence represents zona pellucida-bound ZP3 mAb); and NKG2D^+^ NK cells (red fluorescence) detectable outside and inside the zona pellucida (white arrows). DAPI (nuclear blue staining). Magnification [**(A,B)**: 50×; **(C–F)**: 400×]. Hematolyxin and eosin stain **(A–E)**. [Reproduced from Rival et al. (45), *J. Immunol*. 191, 2865–2869].

ZP3 is a major sperm receptor in fertilization (41). The pZP3 (330–342) contains a pathogenic T cell epitope and a native B cell epitope 335–342 (42), and antibodies to this pZP3 B cell epitope inhibit sperm binding to the zona pellucida (41, 43). Adult mice immunized with pZP3 in CFA develop a pathogenic CD4^+^ T cell response and a non-pathogenic antibody response. The latter was confirmed in adult mice immunized with a chimeric ZP3 peptide that contains the native B cell epitope 335–342 linked to a foreign T cell peptide from the bovine RNAse (43). The mice produce ZP3 antibodies that bind to the ovarian zona pellucida without causing ovarian pathology. However, 70% of the females immunized with the chimeric ZP3 peptide were infertile because of the contraceptive effect of ZP3 antibodies. In the remnant fertile females, the maternal ZP3 autoAb transferred to the neonates induced severe nAOD (Figure 1). Remarkably, nAOD developed in the progeny only when the ZP3 antibody exposure was initiated within the first 6 days of life, as documented by feeding antibody-positive milk to normal pups of different ages, and by antibody transfer (44).

Neonatal autoimmune ovarian disease is a unique and versatile model. *First*, ovaries can be safely removed to deplete ovarian autoantigens; and ovaries from donors of different ages and with different genetic modifications can be implanted under the kidney capsule can be used to monitor the effect of putative molecules in nAOD development. This approach has allowed us to show that nAOD susceptibility is not related to the intrinsic differences between adult and neonatal ovaries (44). It has also allowed us to find, unexpectedly, that nAOD induction requires resident ovarian cells that express FcγRIII. *Second*, nAOD induced by maternal immunization with the novel ZP3 chimeric peptide induces ZP3 antibodies without concomitant pathogenic T cell response. In addition to feeding pups with ZP3 antibody-positive mothers, nAOD is also inducible by passive transfer of a monoclonal antibody that recognizes the pZP3 335–342 (45). This allowed us to adjust the antibody dose to body weight between neonatal or adult mice and show that the nAOD resistance of adult mice is not related to insufficient antibody or ovarian IC. *Third*, there has been uncertainties regarding the physiological relevance of the ZP3 auto-peptide and its cognate antibody because (1) pZP3 was arbitrarily chosen as a novel auto-peptide with T and B cell epitopes; and (2) AOD induction also requires immunization with adjuvants. However, regulatory T cell (Treg) depletion from normal female mice leads to spontaneous production of autoAb that targets the same ZP3 335–342 epitope and the transfer of the sera from these animals induces nAOD in naïve pups. Thus, the pZP3 that has been studied for over 20 years is, in fact, a physiologically relevant B cell autoepitope. *Fourth*, nAOD develops in wild-type mice, and interestingly, nAOD also develops in mice that lack T and B cells. We call them T cell-dependent nAOD (TD-nAOD) model and T cell-independent nAOD (TI-nAOD) model, respectively. TD-nAOD induction is MHC-restricted, and it provides a useful platform for studying neonatal innate and adaptive responses and their interaction. On the other hand, the study of TI-nAOD in the recombination activation gene (Rag) knock out (KO) mice allows a reductionist approach to critically dissect the neonatal innate response. For example, we can define the novel properties of neonatal natural killer (NK) cells *in vivo* without the complex interaction between the innate and adaptive immune cells.

With the two nAOD models, we have addressed two fundamental questions on neonatal autoimmune disease: (1) Why are neonates more susceptible to autoimmunity; and (2) How do maternal autoAb induce an autoimmune disease in neonatal mice?

## Why are Neonates More Susceptible to nAOD?

The neonatal time window of nAOD induction applies equally to TD-nAOD and TI-nAOD. Therefore, neonatal innate responses are sufficient to confer propensity to nAOD in newborn mice (44, 45). NK cells are components of the innate response that can perform antibody-dependent cellular cytotoxicity (ADCC) through FcγRIII and produce multiple cytokines. Differences in the phenotype and function of neonatal and adult NK cells have been described (46). However, while some reports show a poor neonatal NK cell function compared to adults, others demonstrate equal or enhanced effector functions in neonatal NK cells. Our recent work has convincingly demonstrated that neonatal NK cells and their unique properties are the key explanation for newborn propensity to nAOD (45). Strikingly, the age of the donors of NK cells that restore nAOD in genetically NK cell-deficient recipients that lack the rag and the common gamma chain genes, correlated precisely with the neonatal time window for nAOD induction. Thus, neonatal NK cells are critical for nAOD susceptibility (45). NK cell activation depends on the balance of signaling through stimulatory and inhibitory receptors (47). Self-tolerance by NK cells is achieved by the interaction of major histocompatibility complex class I (MHC I) with the murine Ly49 receptors or the human killer cell Ig-like receptors (KIR). However, expression of these receptors is stochastic. Those NK cells that lack receptors for self-MHC I are potentially autoreactive. However, in a process termed licensing, the NK cells that do not recognize self-MHC I become “anergic,” thus ensuring self-tolerance (48). Strikingly, it has been recently shown that the “anergic” NK cells that do not recognize self can become activated during inflammatory conditions and are more efficient in clearing infections or tumor cells than the licensed NK cells (49–51).

**Figure 2:**
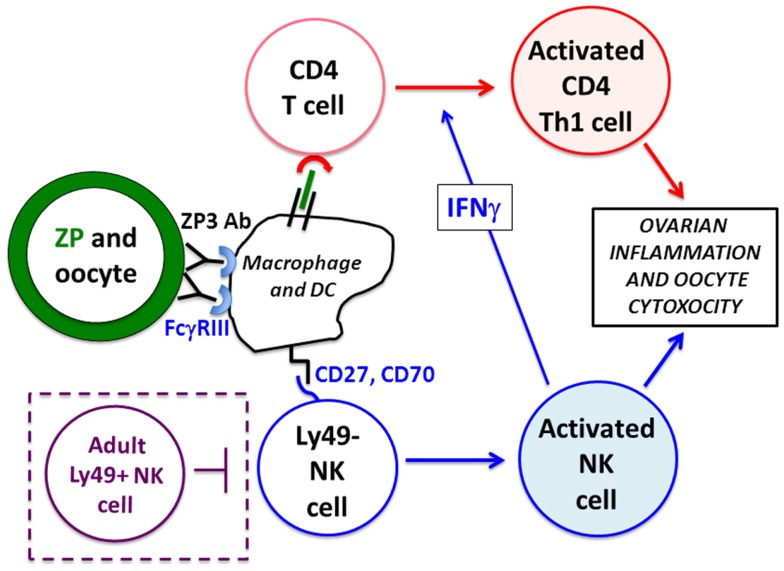
**Mechanism of nAOD induction**. Ovarian ZP3 immune complexes on the zona pellucida (ZP, green) are formed after maternal autoAb transfer and stimulate FcγRIII^+^ macrophage/dendritic cells that activate (1) a *de novo* CD4^+^ T cell response (in red), and (2) Ly49-negative neonatal NK cells (in blue). These NK cells produce IFNγ promoting a Th1 pathogenic CD4^+^ T cell response to ovarian antigens. Ovarian inflammation and oocyte depletion is, in turn, mediated by the FcγRIII^+^ neonatal NK cells and activated Th1 effector CD4^+^ T cells. Mice older than 9 days fail to develop nAOD because of the emergence of Ly49^+^ NK cells (in purple dotted box) and Treg function (not depicted).

The expression of Ly49 receptors is known to be ontogenetically regulated; while ~5% of NK cells express Ly49C/I receptors in the first week of life, the frequency in adult mice is 10 times higher (52–54). This prompted us to investigate the role of Ly49 receptors in nAOD induction, and to address whether the delayed expression of Ly49C/I on NK cells confers adult resistance to nAOD. In a pivotal experiment, we showed that adult Ly49C/I negative NK cells could also induce nAOD only after the Ly49C/I^+^ NK cell subpopulation has been depleted *in vivo*. Our findings made three important points: (1) neonatal NK cells are documented for the first time to be functional and they are, in fact, hyper-reactive, (2) both neonatal and adult Ly49C/I negative NK cells can induce nAOD, and (3) they raise the interesting possibility that adult Ly49C/I^+^ NK cells can inhibit the activation of Ly49C/I negative NK cells, as shown by their capacity to block nAOD induction. Our findings are supported by recent literature on NK cells. Ivarsson et al. (55) recently showed that human fetal NK cells are hyper-responsive to cytokine stimulation, and CD16 engagement can overcome their hypo-responsiveness in killing HLA-negative targets. This study provided a clinical correlate to our findings. In addition, it has been shown that Ly49-negative NK cells can acquire full effector functions under inflammatory conditions. We should emphasize that whereas previous work (49, 51) has described intrinsic NK cell regulation, our study on nAOD provides the first evidence of extrinsic NK cell inhibition, where Ly49C/I^+^ NK cells may inhibit Ly49C/I negative NK cells (Figure 2). However, this possibility still requires further investigation. We currently speculate an indirect regulation that depends on the competition between the two NK cell subsets for the interaction with dendritic cells, which are required for NK cell priming (56, 57).

As a mechanism of nAOD resistance in mice older than 7 days, Treg were documented to control susceptibility to nAOD. Thus, when Treg were depleted from 9-day-old pups with CD25 antibody, the neonatal time window was extended and the older mice became fully susceptible to nAOD (44). Therefore, at least two mechanisms acquired by adult mice confer resistance to autoimmune disease: (1) the innate system, by restraining NK cell activation with the acquisition of the inhibitory Ly49 receptors, and (2) the adaptive system, by acquiring Treg function. In contrast, these mechanisms are deficient in the neonatal mouse.

## How Do the Maternal ZP3 autoAb Cause Ovarian Injury?

### Dual NK cell requirements in nAOD: Induction of antibody-dependent cytotoxicity (ADCC) and ovarian antigen-specific pathogenic CD4 T cell response

Maternal ZP3 autoAb transferred through the milk but not the placenta was critical for nAOD induction (44). Within 24 h, ZP3 IC was detectable in the zona pellucida of the ovary, a process that culminated in significant ovarian inflammation and loss of oocytes over the next 2 weeks [Figure 1; Ref. (44)]. The IC can cause immunopathology by: (1) activation of the complement cascade, (2) FcγRIII-dependent ADCC, and (3) T cell activation mediated by FcγRIII and/or complement receptor-bearing antigen presenting cells (APC). Complement C3b and C5b were undetectable in ovaries of mice with nAOD. However, studies with blocking antibodies and gene KO mice indicated that nAOD is dependent on FcγRIII expression (44, 58). In fact, we found that TD-nAOD pathogenesis requires both FcγRIII-dependent ADCC and a *de novo* T cell response. First, the neonatal NK cell must express FcγRIII to support nAOD (58). Second, the critical CD4^+^ T cells are required in TD-nAOD because (1) T cell depletion prevents nAOD, (2) CD4^+^ T cells from mice with nAOD transfer ovarian disease to naïve pups (44). Importantly, NK cells are required in both the inductive phase and the effector phase of the T cell response (58). Likely, the NK cell-derived IFNγ skews the T cell response toward IFNγ-producing Th1 cells (Figure 2). Indeed, IFNγ neutralization by antibody in the cell donors blocked the adoptive transfer of nAOD (58) and IFNγ-deficient NK cells failed to induce nAOD. Importantly, requirement of NK cells and FcγRIII expression have also been documented in the adult model of myasthenia gravis (59).

In our ongoing research, we have further clarified the pathogenesis of nAOD by making the following observations. First, in addition to NK cells, ovarian resident FcγRIII^+^ macrophages and/or dendritic cells are requisites in TD-nAOD. Second, NK cell activation most likely occurs in the lymph nodes where they are primed by the ovarian-derived FcγRIII^+^ cells. Third, NK cell homing and activation are dependent on IL-15, CD70 and CD27, and CXCR3.

## Antibody to Viral Antigen also Co-Stimulates Active Immunity to Virus in Neonatal but Not Adult Mice

Maternal anti-microbial antibodies confer transient protection from infection to the progeny. However, compelling studies have shown that co-injection of a small amount of antiviral antibody with a live virus in neonatal mice can evoke effective long-term immunity against the virus. Neonatal infection with the FrCasE murine retrovirus before day 5–6 after birth leads to a virus-specific Treg response that inhibits efficient antiviral immunity (60). This results in fatality from neurodegeneration within 2 months and from erythroleukemia within 4–5 months. However, the Treg response is dramatically curtailed when the virus-infected neonatal mice receive a small dose of epitope-specific antiviral antibody within 2 days after the viral infection (61, 62). Concomitantly, the mice develop a strong antivirus CD8^+^ T cell response and life-long protection against the virus. As will be described below, the underlying mechanisms behind this successful neonatal antiviral vaccination schema are remarkably similar to the mechanism involved in nAOD induction. Therefore, neonatal exposure to antibody can enhance immunity against both autologous and foreign antigens.

## Common Mechanisms are Shared between Autoimmunity and Viral Immunity Induction by Neonatal Immune Complexes

It is remarkable that neonatal IC formation by an epitope-specific antibody can lead to both autoimmune disease and antiviral immunity. Even more striking is the fact that the two responses deploy very similar mechanisms. They both require: (1) antibody injection and IC formation within the first week of life, (2) epitope-specific antibody that targets the functional domain of the antigen, (3) NK cells, (4) FcγRIII, (5) *de novo* induction of T cell responses, and (6) IFNγ. In both cases, the responses to IC beyond the neonatal time window were inhibited by Treg. Based on findings from our recent nAOD study, we can now add to this list, the critical influence of NK cell expression of the Ly49 inhibitory receptors.

## General Conclusion/Summary

It is generally accepted that the higher susceptibility of newborns to infections is a consequence of the immature neonatal immune system. In support of this concept, many studies have described a weaker response by the neonatal immune system. However, this concept is still controversial, as others have found an equivalent or even stronger neonatal immune responses over the adult response. Moreover, a recent study showed that the poor protection to respiratory syncytial virus infection in newborns is due to the inhibition of the antibody production by the IFNγ produced by neonatal NK cells and T cells (63), suggesting that the neonatal immune system involves a complex cellular and molecular interplay. Experimental data derived from nAOD research have added new insight on the *in vivo* responsiveness of the newborn immune system. They clearly indicated that neonatal mice have the capacity to mount a robust immune response to tissue-associated IC that surpasses an adult response. The primary mechanism is the lower threshold of neonatal NK cell responses to tissue IC; and the capacity of neonatal NK cells to promote a pathogenic neonatal T cell response. It is important to emphasize that recent studies on human NK cells in support of this concept are also emerging (55). Our findings on nAOD are concordant with the observations by Michaud et al. (61) and Nasser et al. (60, 62) on the neonatal response to a foreign antigen in the context of a viral infection in the newborn. In both, the antibody exposure restricted to the first few days of life has been clearly documented to strongly enhance the neonatal immune response against its cognate antigen. The mechanisms shared between these two models should support a novel approach in effective vaccine design for the very young.

## Conflict of Interest Statement

The authors declare that the research was conducted in the absence of any commercial or financial relationships that could be construed as a potential conflict of interest.
